# Preparation and Application of a Novel Anti-Contamination Agent for Use in Drilling Fluids

**DOI:** 10.3390/ma19122595

**Published:** 2026-06-16

**Authors:** Song Zhang, Xi Guan, Fei Deng, Xiaowei Cheng

**Affiliations:** 1 Chuanqing Drilling Engineering Co., Ltd., Drilling Fluid Technology Services Company, Chengdu 610051, China; zhangsongtd@126.com; 2 School of New Energy and Materials, Southwest Petroleum University, Chengdu 610500, China; gx173112506081@outlook.com (X.G.); 202322001165@stu.swpu.edu.cn (F.D.); 3 Key Laboratory of Oil and Gas Reservoir Geology and Exploitation, Southwest Petroleum University, Chengdu 610500, China

**Keywords:** anti-contamination agent, drilling fluid, spacer fluid, layered double hydroxide (LDH), compatibility

## Abstract

**Highlights:**

This article uses the co-precipitation method to prepare a zinc aluminum hydrotalcite precursor and then intercalates ATMP into the interlayer of hydrotalcite by the ion exchange method to prepare Zn/Al TMP LDH. Through FT-IR, XRD, and TG-DTG analysis of Zn/Al ATMP LDH, it was found that ATMP was successfully inserted into the interlayer of the water slide layer, and the anti-pollution agent was successfully prepared. By testing the compressive strength, it was found that compared with directly adding ATMP (with a 1-day strength of 0 MPa at 0.3 wt%), Zn/Al TMP LDH had a 1-day strength of 32.84 MPa at the same dosage, and its 7-day strength continued to increase. The anti-pollution isolation fluid had good compatibility with drilling fluid and cement slurry in different mixing ratios (7:3, 5:5, and 3:7). Based on the adsorption properties of hydrotalcite materials and the chelating ability of ATMP, a synergistic anti-pollution mechanism of “adsorption regulation–stable dispersion–sustained release” was proposed through the above experimental results. Moreover, the anti-pollution isolation fluid was successfully applied in cementing operations under extreme working conditions of 187 °C × 145 MPa, providing new materials and technical solutions for deep and ultra-deep well cementing construction.

**What are the main findings?**

- Preparation of a high-efficiency anti-fouling agent, Zn/Al-ATMP-LDH, by the intercalation method.
- The anti-pollution agent has good compatibility and can effectively improve the pollution problem of drilling fluid caused by pollution sources.
- The configured isolation fluid has excellent performance and was successfully applied in high-friction blocks.

**What are the implications of the main findings?**

- The development of anti-pollution agents can adopt the approach of combining a polymer and nanotechnology to resolve the long-standing contradiction between existing ‘anti-pollution agents’ and ‘slow strength development’.
- Future research can focus on establishing a unified mechanistic model around interfacial adsorption, ion complexation, particle dispersion, and functional slow release to guide the design of inhibitors for different pollution systems (oil-based, water-based, and high-density drilling fluids).

**Abstract:**

An anti-contamination agent (Zn/Al–ATMP–LDH) was synthesized by intercalation and used to correct the abnormal thickening and related operational risks caused by contact contamination between drilling fluids and cement slurries during high-temperature/high-pressure cementing. The experimental results show that the agent is chemically stable and exhibits good compatibility with conventional spacer fluid additives. When compared with the direct addition of amino tris(methylenephosphonic acid) (ATMP), confining ATMP within a layered double hydroxide (LDH) markedly mitigates the retarding effect. At a dosage exceeding 0.3 wt%, the compressive strength of cement stone increased from 0 to 32.84 MPa following curing at 90 °C for 1 day and continued to develop steadily after 7 days. Following conditioning at 187 °C and 145 MPa for 120 min, the spacer system formulated using the proposed agent as the core component served to enhance the rheology of the mixed slurry via synergistic adsorption–regulation–dispersion stabilization-controlled release. The mixed slurry maintained stable rheological properties before and after aging with no uncontrolled thickening. When mixing the cement slurry and drilling fluid at a 7:3 volume ratio, the slurry consistency exceeded 60 Bc within 1 h, failing to meet operational requirements. In contrast, the mixed slurry containing the anti-contamination spacer (cement slurry–drilling fluid–spacer = 7:2:1) exhibited a thickening time greater than 300 min and was successfully applied in field-cementing operations in a well in the Gaomo area.

## 1. Introduction

With the development of deep, ultra-deep, and high-temperature/high-pressure (HTHP) oil and gas resources, oil-based drilling fluids (OBDFs) have been widely used in complex formations due to their excellent inhibitive capacity, lubricity, and thermal stability [1,2,3,4]. The application of OBDFs is critical for maintaining wellbore stability and reducing downhole accident risks in HTHP wells and complicated hole sections. However, during casing running and cementing operations, contact between drilling fluid and cement slurry is unavoidable, leading to severe contamination. This issue has become a key technical challenge that threatens the safe and stable application of OBDFs [5].

When cement slurry contacts drilling fluid, the invasion of free water and the migration of Ca^2+^ and OH^−^ from cement hydration disrupt the invert emulsion structure of OBDFs. Studies have shown that Ca^2+^ can react with emulsifiers to reduce electrical stability [6,7,8], while OH^−^ alters the wettability of barite, resulting in severe rheological deterioration [9,10]. Although recent efforts have attempted to quantify this contamination under static conditions [11,12], the dynamic chemical interference under HTHP conditions—where contamination effects are most pronounced—remains insufficiently understood. Therefore, the development of efficient and stable anti-contamination technologies is essential to ensure drilling safety in HTHP wells.

To mitigate contact contamination, three main strategies have been explored in the literature. (1) Adjustment of emulsifier systems: Some studies propose increasing emulsifier dosage or adding wetting agents to stabilize the invert emulsion [13,14]. While effective at low contamination levels, this approach often fails under HTHP conditions due to emulsifier degradation [15]. (2) Polymeric dispersants and chelating agents: Free phosphonate additives (e.g., ATMP) have been used to chelate Ca^2+^, thereby delaying cement hydration [16,17]. However, a critical drawback is the severe retardation of cement strength development [18,19,20]. As demonstrated in Section 3.2 of this work, free ATMP reduces 1-day compressive strength to 0 MPa at dosages > 0.3 wt%. (3) Nanomaterials: Recent advances have highlighted the potential of layered double hydroxides (LDHs) for ion adsorption and controlled release in cementing applications [21,22,23]. Nevertheless, current LDH-based additives have primarily been designed for fluid loss control rather than for managing chemical contamination between incompatible fluids [24]. Furthermore, the intercalation of phosphonate inhibitors into LDH interlayers for slow-release anti-contamination purposes has not been reported. Consequently, a critical trade-off persists between achieving immediate contamination control and maintaining long-term strength development in cement–drilling fluid mixtures.

Layered double hydroxides (LDHs) exhibit positively charged host layers, exchangeable interlayer anions, a high specific surface area, and structurally tunable frameworks [25]. These features enable LDHs to adsorb and immobilize anionic contaminants, regulate ionic strength, and stabilize dispersed systems [26,27]. To address the aforementioned challenges, this study proposes a novel anti-contamination agent based on a Zn–Al LDH structure, into which amino tris (methylenephosphonic acid) (ATMP) is intercalated via a two-step coprecipitation and ion-exchange method [28]. Unlike free ATMP, the intercalated structure is designed to provide controlled release of phosphonate groups, thereby mitigating the retardation of cement hydration while maintaining anti-contamination efficacy. More importantly, when benchmarked against other LDH-based systems previously reported for wellbore applications (e.g., unmodified Zn/Al–NO_3_–LDH or LDH used solely as fluid loss additives [24,29,30,31,32,33]), our material offers a dual-function synergistic effect. Unmodified LDHs can provide physical adsorption and nucleation sites but lack the specific chemical functionality to chelate multivalent ions (Ca^2+^/Mg^2+^), which are the primary drivers of contamination. By intercalating ATMP, our system combines the thermal stability and adsorptive capacity of LDH with the precise ion-control capability of phosphonates. This synergy is absent in previously reported LDH additives, which have been designed primarily for fluid loss control or as rheological modifiers, not for managing chemical contamination at the cement–drilling fluid interface.

The proposed anti-contamination agent is hypothesized to regulate the migration of Ca^2+^ and OH^−^ via an ion-exchange and synergistic release mechanism when the cement slurry comes into contact with the drilling fluid, thereby impeding the chemical interference of the cement slurry with the OBDF system [14,15,16,17,18,19]. The findings of this study offer a new material design strategy and a mechanistic understanding of contamination control in OBDFs. Moreover, the developed agent has been successfully applied in field operations in a well located in the Gaomo block.

## 2. Materials and Methods

### 2.1. Materials

The materials used in this study included Jiahua G-grade oil well cement, quartz sand, iron ore powder, silica fume, barite, and composite whiskers. The additives used in the oil well cement system were SDP-1 (dispersant) [Guanghan, China], SD210 (retarder) [Guanghan, China], SD130 (fluid loss agent) [Guanghan, China], SD52 (defoamer) [Guanghan, China], SD77 (strength enhancer/anti-gas channeling agent) and SD35 (suspension stabilizer) [Guanghan, China]. The cement slurry formulation was as follows: 100 wt% Jiahua G-grade cement + 31.7 wt% quartz sand + 1.12 wt% iron ore powder + 2.43 wt% silica fume + 2.43 wt% SDP-1 + 4.86 wt% SD77 + 1.46 wt% SD35 + 7.31 wt% composite whiskers + 4.39 wt% SD210 + 12.19 wt% SD130 + 0.48 wt% SD52 + 63.9 wt% water; the slurry had a density of 2.32 g/cm^3^. The oil-based drilling fluid was collected from an on-site drilling operation in the Sichuan–Chongqing region. The fluid composition and properties were as follows: 0.2% sand content; 30% solids content; 4% oil-phase content; 17.16 g/L bentonite; 38,000 mg/L Cl^−^; 400 mg/L Ca^2+^; 8% plugging agent content; and density = 2.28 g/cm^3^.

The materials used in the preparation of the anti-contamination agent included amino tris(methylenephosphonic acid) (ATMP) [Chengdu, China], zinc nitrate hexahydrate (Zn(NO_3_)_2_·6H_2_O) [Chengdu, China], aluminum nitrate nonahydrate (Al(NO_3_)_3_·9H_2_O) [Chengdu, China], sodium hydroxide (NaOH) [Chengdu, China], sodium nitrate (NaNO_3_) [Chengdu, China], and ethylene glycol. All of the chemicals were purchased from Chengdu Kelong Chemical Reagent Factory and were of analytical grade. The CAS numbers of the materials used in this experiment are listed in Table 1 and Table 2. 

### 2.2. Experimental Method

The cement slurries were prepared in accordance with GB/T 19139–2012 (Test Methods for Oil Well Cement) [34]. Mixed slurry compatibility tests were conducted using a high-temperature/high-pressure consistometer (TG-8040DA) [Mettler Toledo Instrument Co., Ltd., Greifensee, Switzerland; SDTA85], following the thickening-time procedure specified in the standard. Thickening tests were conducted by mixing the cement slurry, drilling fluid, and spacer fluid at different mass ratios using an additional high-temperature/high-pressure consistometer (OWC-9308, Institute of Applied Technology, Shenyang Institute of Aeronautical Industry) [Shenyang, China]. The mixed slurries were cured in an atmospheric consistometer (OWC-9350A, Shenyang Aeronautical Research Institute, Shenyang, China). Rheological properties before and after curing were assessed using a six-speed rotational viscometer (ZNN-06B, Qingdao Tongchun Petroleum Instrument Co., Ltd., Qingdao, China). All rheological tests after high-temperature aging were carried out immediately after the high-temperature and high-pressure curing/hot-rolling aging of the sample was completed, and it was taken out and cooled to room temperature, stabilized for 10 min, and then immediately carried out. Rheological testing was completed within 30 min to ensure data consistency and comparability. The compressive strength of the cured cement stone was determined using a compression testing machine (TYE-300, Jianyi Instrument Machinery Co., Ltd., Wuxi, China).

An X-ray diffractometer (DX-2700, Dandong Haoyuan Instrument Co., Ltd., Dandong, China) was employed to identify the characteristic diffraction peaks of the hydrotalcite-like material and confirm successful synthesis. Fourier-transform infrared spectroscopy (FT-IR, WQF-520, Beijing Rayleigh Analytical Instrument Co., Ltd., Beijing, China) was utilized to identify the functional groups associated with the hydrotalcite precursor and the intercalated hydrotalcite-type anti-contamination agent in order to verify successful incorporation of the anti-contamination molecules in the interlayer structure. The particle size of the synthesized anti-contamination agent was measured using a laser particle size analyzer (Mastersizer 2000, Malvern Instruments Ltd., Worcestershire, UK). Thermogravimetric–differential thermogravimetric analysis (TG-DTG, SDTA85, Mettler Toledo, Greifensee, Switzerland) and scanning electron microscopy (SEM, ZEISS EVO MA15, Carl Zeiss, Oberkochen, Germany) were used to evaluate the thermal stability and microstructural morphology of the anti-contamination agent.

### 2.3. Preparation of the Anti-Contamination Agent

Firstly, 18.73 g of Zn(NO_3_)_2_·6H_2_O and 7.87 g of Al(NO_3_)_3_·9H_2_O were dissolved in deionized water and diluted to a final volume of 200 mL. The resulting solution is denoted as solution M. In addition, 17.00 g of NaNO_3_ was dissolved in 250 mL of deionized water. When the solution had cooled, 16.00 g of NaOH was slowly added until fully dissolved, and the final volume was adjusted to 400 mL, yielding mixed solution B that contained 1.0 mol/L NaOH and 0.5 mol/L NaNO_3_.

Next, 200 mL of deionized water was added to a three-neck flask and heated to 70 °C. Solutions M and B were simultaneously added dropwise into the flask. The pH of the system was continuously monitored during co-precipitation and maintained in the 9.5–10 range. The coprecipitation reaction proceeded for 7 h. The suspension was cooled to room temperature, vacuum-filtered, and washed with deionized water until neutral. The resulting solid was freeze-dried and then dried in an oven at 60 °C for 24 h. After grinding, a white powder (Zn–Al–NO_3_–LDH) was obtained.

The ATMP was dissolved in deionized water, and the pH of the solution was adjusted to 9 with NaOH. A known amount of Zn/Al–NO_3_–LDH was dispersed in the ATMP solution and stirred at 55 °C for 5 h, allowing a gradual replacement of interlayer NO_3_^−^ ions by ATMP through intercalation. Following the reaction, the solid product was separated by centrifugation, repeatedly washed with deionized water until neutral, dried in an oven, and then ground and sieved to obtain the anti-contamination agent (Zn/Al–ATMP–LDH).

**Figure 1:**
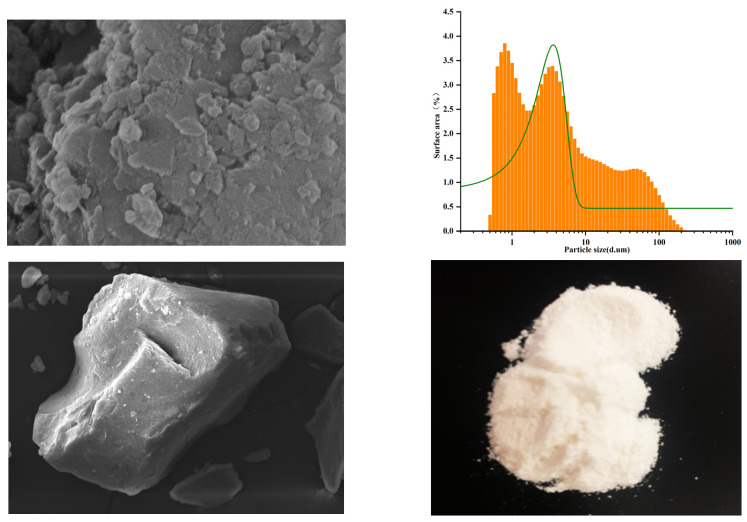
Morphology and particle size distribution of the anti-contamination agent.

As shown in Figure 1, the prepared anti-contamination agent exhibited a layered stacked structure with clear particle boundaries and relatively smooth edges. The particle size analysis indicated that the majority of particles were distributed in the 1–10 μm range, with D(10), D(50), and D(90) values of 0.705 μm, 3.221 μm, and 42.160 μm, respectively. indicating that the anti-contamination agent was principally composed of fine micro/nanoscale particles.

## 3. Results and Discussion

### 3.1. Preparation and Characterization of the Anti-Contamination Agent

#### 3.1.1. FT-IR Analysis

**Figure 2:**
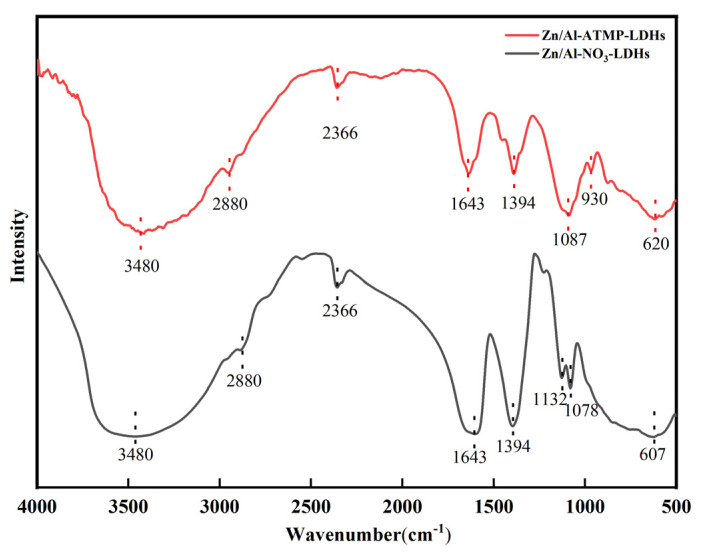
FT-IR spectra of the anti-contamination agent.

The FT-IR spectra of the anti-contamination agent and precursor are shown in Figure 2. It can be seen that both samples exhibit absorption features that are typical of LDH materials. The broad band at 3480 cm^−1^ is assigned to the O–H stretching vibration of hydroxyl groups in the host layers and interlayer water molecules, whereas the band at 1643 cm^−1^ corresponds to the H–O–H bending vibration of water molecules. The absorption band in the 607–620 cm^−1^ range is attributed to Zn/Al–O framework vibration, indicating that the layered double hydroxide structure is stable.

In the case of Zn/Al–NO_3_–LDH, characteristic nitrate absorption bands were recorded at 1394 and 620 cm^−1^, and relatively strong absorption peaks are also observed near 1132 and 1078 cm^−1^, confirming the intercalation characteristics of the inorganic anion system. In contrast, the spectrum of Zn/Al–ATMP–LDH shows strong characteristic P–O vibration bands at 1087 and 930 cm^−1^, whereas the characteristic peak at 1394 cm^−1^ is significantly weaker. These changes serve to indicate that ATMP was successfully introduced into the interlayer space and exchanged with NO_3_^-^. The observed variation in the characteristic peaks confirms that Zn/Al–NO_3_–LDH was effectively modified by ATMP ion-exchange intercalation.

#### 3.1.2. XRD Analysis

**Figure 3:**
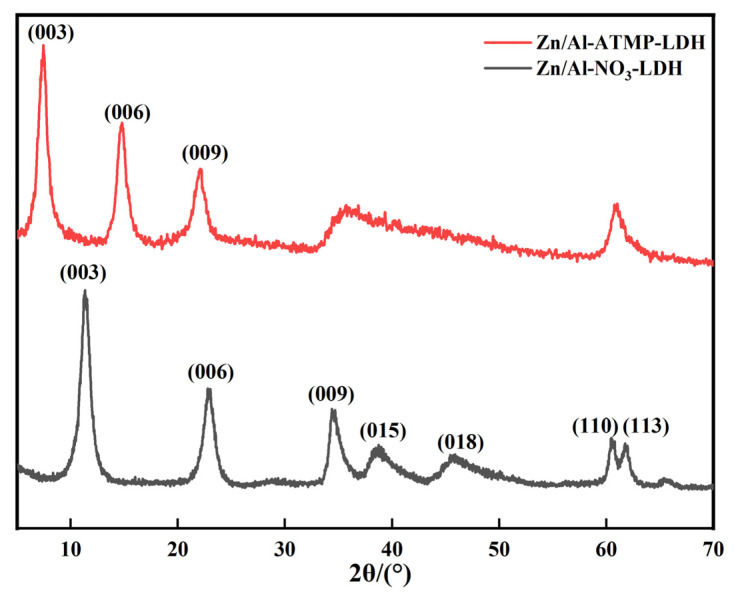
XRD patterns of the anti-contamination agent and precursor.

The XRD patterns of the anti-contamination agent and precursor are presented in Figure 3. In the case of Zn–Al–NO_3_–LDH, a series of basal reflection peaks, including (003), (006), and (009), appear in the low-angle region. In addition, non-basal diffraction peaks, such as (015), (018), and the (110) and (113) reflections near 60°, are present in the medium- and high-angle regions. These results indicate that the sample possesses a well-ordered layered stacking structure and good crystallinity, which are characteristic features of the LDH phase.

When compared with Zn–Al–NO_3_–LDH, Zn–Al–ATMP–LDH retained the basal (003), (006), and (009) reflections, but the associated peaks were shifted to lower diffraction angles. This shift can be attributed to the replacement of the relatively small interlayer NO_3_^−^ ions by ATMP containing multiple phosphonic acid groups, which serve to significantly expand the interlayer spacing. In addition, the intensities of the non-basal (015) and (018) peaks were markedly weakened and even difficult to distinguish following ATMP modification. This suggests that the intercalation process lowered the stacking order of the LDH layers, decreasing the crystallite size. These changes further confirm that ATMP was successfully inserted into the interlayer galleries.

#### 3.1.3. Thermal Stability

**Figure 4:**
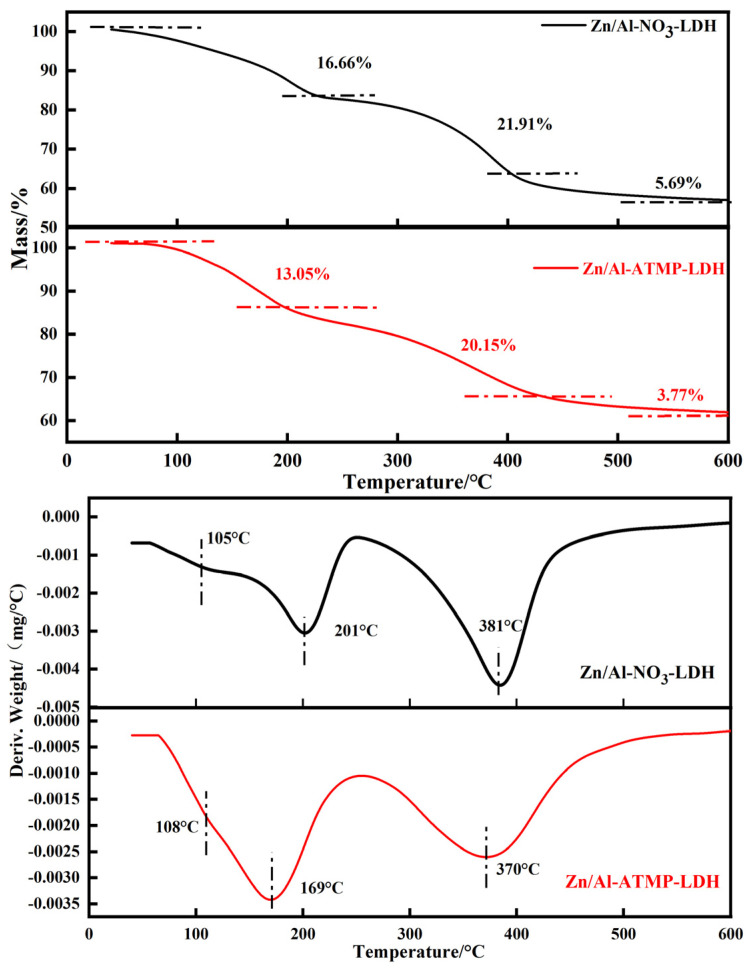
TG-DTG profiles for the anti-contamination agent.

The thermogravimetric results for the anti-contamination agent and precursor are shown in Figure 4. Both samples exhibit the typical thermal response associated with LDH materials. The Zn/Al–NO_3_–LDH weight loss peaks, at approximately 105, 201, and 381 °C, correspond to the removal of interlayer water (16.66%), dehydroxylation of the host layers and decomposition of interlayer NO_3_^−^ anions (27.61%), which led to the collapse of the layered structure.

The low-temperature weight loss for Zn/Al–ATMP–LDH decreased by 3.61%, indicating that the intercalation of ATMP altered the water-binding characteristics, reducing the amount of bound water. In addition, the weight loss peaks in the medium- and high-temperature regions were broadened and shifted slightly to lower temperatures, suggesting that intercalation serves to reduce the stacking order of the layers, increasing the microstrain in the structure so that dehydroxylation and ATMP release occur more readily. The total weight loss following ATMP intercalation decreased from 44.26% to 36.97%, where the weight loss in the high-temperature region decreased from 5.69% to 3.77%. These results indicate that the introduction of ATMP improves stability at elevated temperatures.

### 3.2. Effect of the Anti-Contamination Agent on the Compressive Strength of Cement Stone

**Figure 5:**
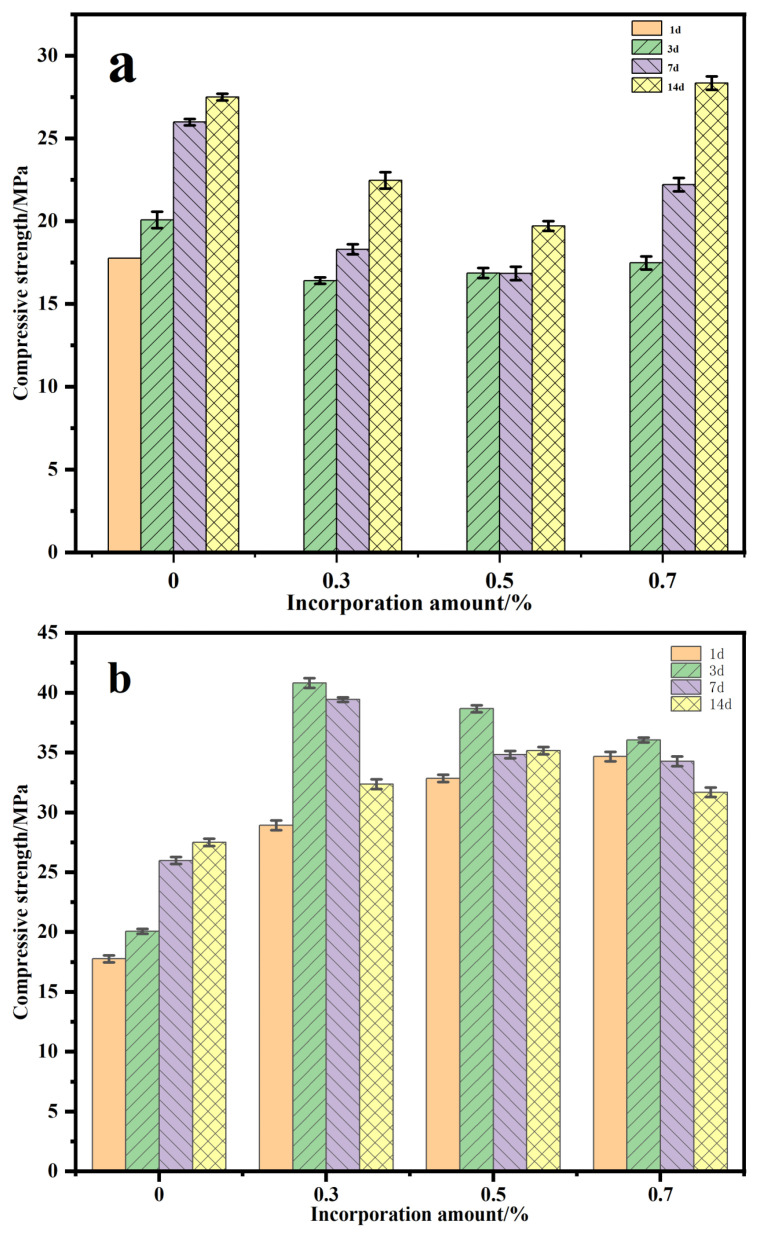
Cement stone compressive strength analysis: (**a**) temporal variation in strength with the direct addition of ATMP; (**b**) temporal variation in strength following the incorporation of Zn/Al-ATMP-LDH.

Cement slurries containing different dosages of the agent were cured at 90 °C to evaluate the effect of the anti-contamination agent on cement stone; the results are presented in Figure 5. It is evident that the incorporation of ATMP markedly inhibited the early compressive strength development. When the dosage exceeded 0.3 wt%, the compressive strength following curing at 90 °C for 1 day dropped to 0 MPa. However, it should be noted that the measured strength values exhibited a standard deviation of approximately ±1.2–2.5 MPa across three replicate samples (see error bars in Figure 5a), indicating some variability in cement hydration kinetics. This variability may be attributed to minor differences in the dispersion homogeneity of the additive within the cement slurry, as well as inherent fluctuations in the curing temperature (±1.5 °C) during the 1-day curing period. Although the strength shows a slight recovery after 7 days of curing, it still fails to meet field application requirements. This response indicates that free ATMP exerts a strong retarding effect with respect to cement hydration.

In contrast, the incorporation of Zn/Al–ATMP–LDH served to significantly enhance strength development. The early compressive strength of cement stone increased from 17.76 MPa to 28.92–34.66 MPa, where the maximum value (40.81 MPa) was attained after 3 days of curing. Moreover, following curing for 7 days, the compressive strength of all Zn/Al–ATMP–LDH-containing samples is higher than that of the neat cement slurry. These results suggest that the intercalation of ATMP in the LDH structure reduces the free-state concentration. During cement hydration, ATMP is gradually released as opposed to a sudden direct contact with the cement slurry, weakening Ca^2+^ complexation during hydration. As the prepared Zn/Al–ATMP–LDH particles are formed at the nanoscale, this provides more nucleation sites and fills the pores in the cement stone, promoting hydration products with microstructure densification. As a result, the material exhibits a pronounced increase in early strength and a stable increase in strength at later curing ages.

The compressive strength enhancement observed with Zn/Al–ATMP–LDH can be contextualized by comparing it with previously reported anti-contamination agents and cement additives. Free phosphonate additives (e.g., ATMP and HEDP) typically cause severe retardation of early cement strength. For instance, Li et al. [21] reported that the addition of 0.2 wt% ATMP reduced the 1-day compressive strength of Class G cement by approximately 85% at 90 °C. Similarly, Huang et al. [17] observed that conventional chelating agents delayed strength development beyond 7 days, with 1-day strengths often falling below 5 MPa. In contrast, our Zn/Al–ATMP–LDH additive achieved a 1-day compressive strength of 32.84 MPa at a dosage of 0.3 wt% (equivalent to approximately 0.06 wt% ATMP equivalent loading). This value is comparable to or exceeds that of recently reported nanomaterial-based additives. For example, Gautam et al. [16] reported a 1-day strength of 28.5 MPa for nano-silica modified cement at 90 °C, while Arbad, N et al. [26] achieved 31.2 MPa using a polymer-based anti-contamination agent. However, unlike our material, these previous additives did not simultaneously address contact contamination between drilling fluid and cement slurry. Therefore, Zn/Al–ATMP–LDH offers a unique combination of contamination control and early strength development that has not been previously demonstrated.

### 3.3. Compatibility of the Anti-Contamination-Agent-Containing Spacer Fluid Under Different Conditions

#### 3.3.1. Compatibility of the Anti-Contamination Spacer Fluid

Table 3 shows the experimental plan and conditions for evaluating the compatibility of anti pollution agents. The prepared agent was combined with other spacer fluid additives to test chemical stability in formulating an anti-contamination spacer fluid. The changes to the rheological properties of the spacer fluid were tested after aging at 187 °C and 145 MPa for 3 h; the results are presented in Table 4 and Table 5. The experimental data establish that stable viscometer readings and rheological parameters were obtained for all the systems before and after aging, with no obvious thickening or gelation. This indicates that after compounding with different dispersants, fluid-loss additives, and suspension stabilizers, the anti-contamination agent maintains an overall controllable rheology response with no evidence of incompatibility.

**Figure 6:**
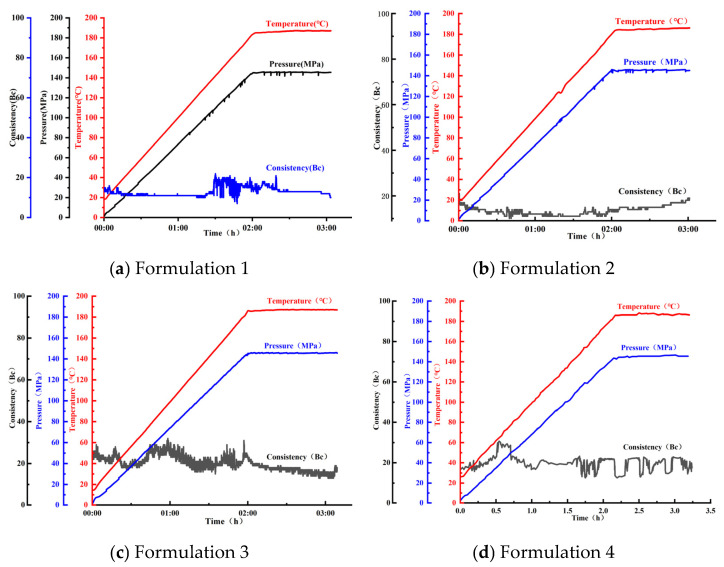
Spacer fluid curing curves.

Figure 6 shows the curing curve of the isolation liquid. Although the consistency of different spacer fluid formulations fluctuated during aging, the associated curing curves exhibit a gradual decline with increasing aging time. A comparison of the apparent viscosity and yield point of the spacer fluids before and after aging demonstrates that the aged spacer fluids retain suitable pumpability and structural stability. The results confirm that the anti-contamination agent shows good compatibility with other spacer fluid additives.

#### 3.3.2. Compatibility of the Anti-Contamination Spacer Fluid and Drilling Fluid at Different Temperatures

**Figure 7:**
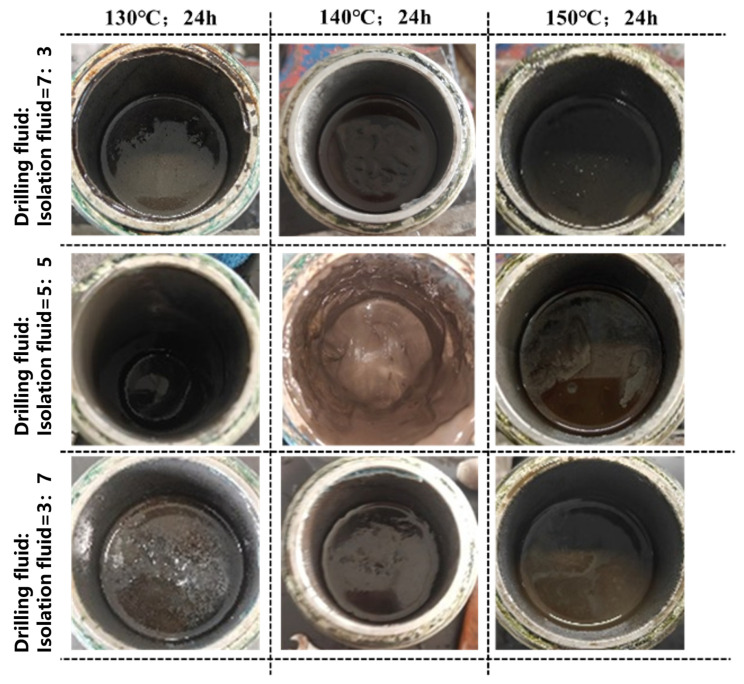
Images showing the changes in morphology for the mixed-slurry section after aging.

The rheological parameters of mixed slurries composed of drilling fluid and spacer fluid at different mixing ratios following aging at 50–150 °C for 24–48 h are presented in Table 6, Table 7 and Table 8. The results indicate that the mixed-slurry systems exhibit pseudoplastic behavior, where widespread irreversible thickening or solidification was not observed with increasing aging temperature or time. This response demonstrates that, following the incorporation of the anti-contamination agent, the spacer fluid shows good compatibility with the drilling fluid. At a drilling fluid-to-spacer fluid ratio of 7:3, the flow behavior index (n) under all the tested conditions falls within the 0.50–0.82 range, indicating suitable pumpability of the mixed slurry. When the drilling fluid-to-spacer fluid ratio is 3:7, the overall viscosity of the mixed slurry is higher, suggesting that increasing the proportion of spacer fluid enhances the carrier and suspension capacity of the system. Moreover, the slurry shows fluctuation patterns associated with shear-thinning under different aging conditions. In contrast, at a drilling fluid-to-spacer fluid ratio of 5:5, the mixed slurry is most sensitive to aging temperature and time, and abnormal thickening occurs after aging at 150 °C for 24 h (Figure 7). This effect is significantly alleviated when the aging is extended to 48 h, indicating that the thickening is reversible.

The results suggest that the observed compatibility of the mixed slurry after the addition of the anti-contamination agent may result from synergistic effects. The positively charged LDH layers can adsorb and capture anionic polymers, clay particles, and contaminating ions in the drilling fluid, impacting polymer bridging and particle flocculation. In addition, the ATMP groups can chelate multivalent metal ions such as Ca^2+^ and Mg^2+^, suppressing “salting-out” and crosslinking-associated thickening due to the changes in ion concentration. The suspension-stabilizing components and weighting solids in the spacer fluid work in tandem to maintain a low-shear structural strength, preventing incompatibility features such as persistent high viscosity and high yield stress. The proposed anti-contamination spacer fluid system exhibits good rheological stability and compatibility with the field drilling fluid over a range of mixing ratios and under high-temperature aging conditions.

The rheological stability of our anti-contamination spacer fluid under HTHP conditions (187 °C; 145 MPa) compares favorably with previously reported systems. Most published spacer fluids are evaluated at temperatures below 150 °C and pressures below 100 MPa [18,22,27]. For example, Henry et al. [13] reported a methyl ester sulphonate spacer fluid that maintained stable viscosity up to 120 °C, but experienced significant thickening above 140 °C. Similarly, the surfactant-based spacer fluid developed by Liu et al. [22] showed good compatibility at 130 °C but exhibited a 40% increase in apparent viscosity after aging at 150 °C for 24 h. In our system, the apparent viscosity of Formulation 2 increased by only 8.5% (from 40.5 to 68.0 mPa·s) after aging at 150 °C for 24 h, and actually decreased slightly after 48 h of aging, suggesting reversible thickening behavior. This reversible response is distinct from the irreversible thermal degradation reported in most previous studies [15,20], and may be attributed to the controlled release of ATMP from the LDH interlayers.

### 3.4. Effect of the Anti-Contamination-Agent-Containing Spacer Fluid on the Thickening Time of the Cement Slurry and Drilling Fluid at Different Temperatures and Pressures

**Figure 8:**
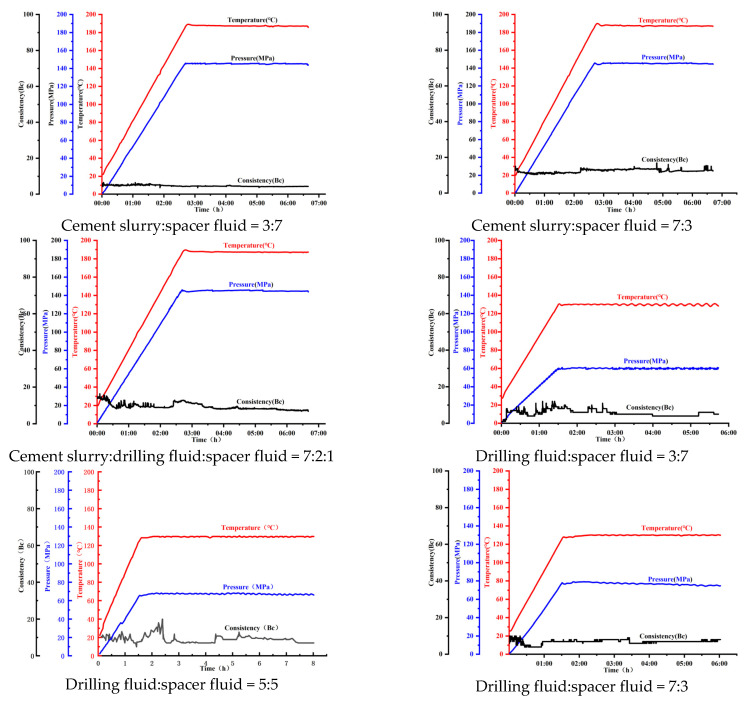
Thickening curves for the mixed-slurry section at different volume ratios of cement slurry, drilling fluid, and spacer fluid.

The anti-contamination performance of the spacer fluid after contact with the cement slurry and drilling fluid was evaluated under actual operating conditions. The anti-contamination spacer fluid was mixed separately with the drilling fluid and cement slurry at different volume ratios. Thickening tests were conducted at 187/128 °C and 145/60 MPa to determine if the mixed-slurry section can meet the thickening-time requirements for field operations. The results are shown in Figure 8, where it is evident that none of the mixed-slurry systems exhibited a sustained rapid increase or uncontrolled rise in consistency under the high-temperature/high-pressure conditions. The consistency remained low and fluctuated smoothly, with no evidence of undesirable flash setting or false setting in the mixed-slurry section. The results demonstrate that the anti-contamination spacer fluid exhibits good compatibility with the drilling fluid and the cement slurry. At a cement slurry:drilling fluid:spacer fluid volume ratio of 7:2:1, the consistency showed some fluctuation during the heating stage and then stabilized, with no flash setting or persistent abnormal thickening. When the drilling fluid:spacer fluid ratio was 5:5, a slight increase in consistency was observed in the mixed-slurry section, but the consistency subsequently reached a stable range, which is consistent with the results discussed above.

The thickening times of all the mixed-slurry systems exceed 300 min, which satisfies the requirements for safe field operation. These results confirm that, following the incorporation of the Zn/Al–ATMP–LDH anti-contamination agent, the spacer fluid can effectively suppress irreversible thickening caused by ionic disturbances and particle flocculation during mixing. The system maintained good compatibility and operational control under high-temperature/high-pressure conditions.

### 3.5. Mechanistic Features of the Anti-Contamination Agent

We hypothesize that the Zn/Al–ATMP–LDH anti-contamination agent suppresses contact contamination between the cement slurry and drilling fluid via a synergism that involves adsorption, regulation, dispersion stabilization, and controlled release. One plausible explanation is that the positively charged LDH layers possess a high anion-exchange capacity and surface adsorption capability, which enables preferential adsorption of contaminating components in the drilling fluid, including anionic polymers, clay particles, and multivalent anions. This impacts polymer bridging and particle flocculation, reducing the risk of flash setting and abnormal thickening in the mixed-slurry section due to flocculation and agglomeration. Moreover, it is reasonable to infer that the intercalated ATMP contains phosphonic acid groups that can effectively chelate multivalent metal ions such as Ca^2+^ and Mg^2+^, alleviating abnormal thickening caused by sudden changes in ion concentration during mixing.

As a nanostructured material, Zn/Al–ATMP–LDH can provide nucleation sites and exert a filling effect in the system. When compared with free ATMP, the interlayer-confined ATMP exhibits a significantly reduced retarding side effect on cement hydration. Consequently, the early compressive strength of the cement stone is enhanced, and the later strength development is also maintained. In addition, the anti-contamination agent can be effectively compounded with common spacer fluid additives such as dispersants and suspension stabilizers and displays high chemical stability. The spacer-fluid system can maintain pseudoplastic flow behavior and suitable pumpability under high-temperature/high-pressure conditions.

## 4. Field Application

**Figure 9:**
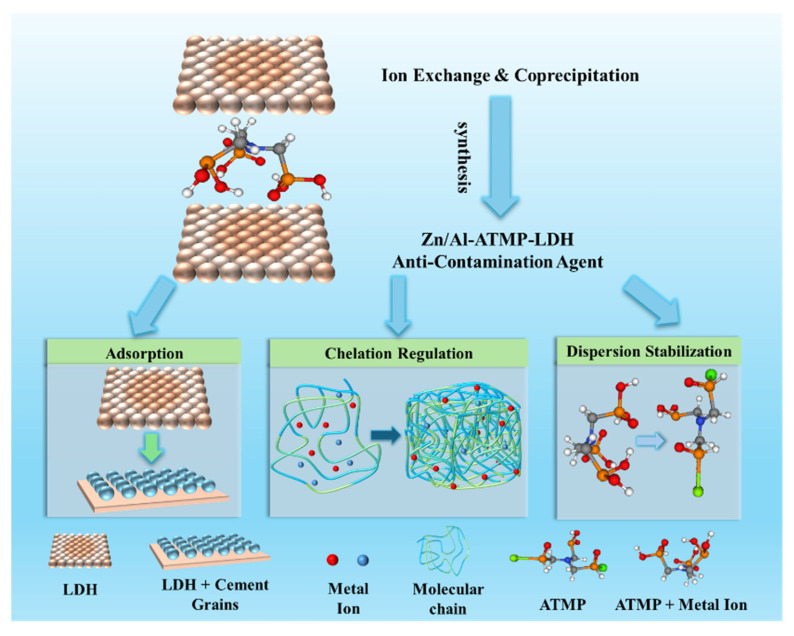
Proposed anti-contamination mechanism.

**Figure 10:**
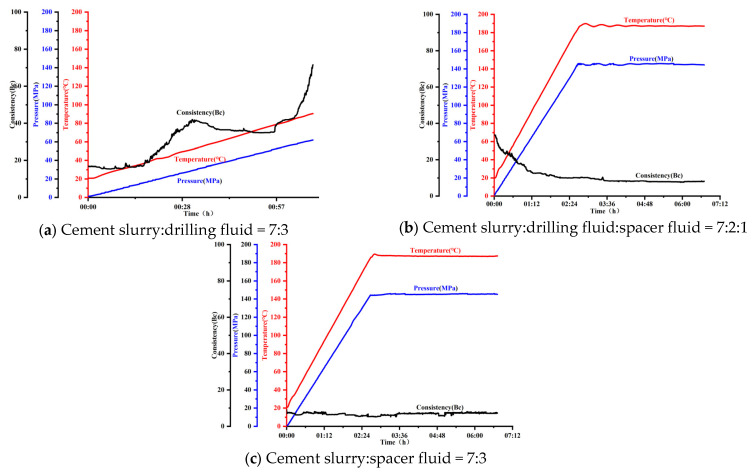
Field contamination experimental results.

The anti-contamination spacer fluid formulated with Zn/Al–ATMP–LDH as the core component was applied in a liner-hanger cementing operation in a well located in the Gaomo area. The well was drilled with a Φ 149.2 mm drill bit to 6373.95 m (after plugging, the artificial bottom hole was 6160.00 m), and it was planned to use a Φ 127 mm tail pipe cementing to isolate the open hole formation and create conditions for safe production in the next step. The on-site drilling fluid was an oil-based drilling fluid containing potassium polysulfide, plugging agent, barite, etc. One of the difficulties in cementing this well is severe contamination and poor compatibility between the drilling fluid and cement slurry. As shown in Figure 9, when the drilling fluid comes into direct contact with the cement slurry, the pollution process curve exhibits an abnormal phenomenon of core wrapping, and its thickening time is severely reduced to only 60 min. The field drilling fluid was oil-based, containing potassium-bearing sulfonates, plugging agents, barite, and other components. One of the major cementing challenges in this well is the severe contact contamination and poor compatibility between the drilling fluid and cement slurry. The field spacer fluid formulation was water + 4% S1 + 0.6% S2 + 0.6% S3 + 5% anti-contamination agent + 5% D1 + 10% micro-manganese + 48% barite. The operating temperature, pressure, and time were 187 °C, 145 MPa, and 120 min. Thickening tests were carried out on mixed slurries with volume ratios of cement slurry:drilling fluid:spacer fluid = 7:2:1 and cement slurry:drilling fluid = 7:3. The results are shown in Figure 10, where it can be seen that when the cement slurry came into direct contact with the drilling fluid, the slurry consistency exceeded 60 Bc within 1 h, and the thickening curve exhibits a stepwise increase. The thickening time is far below the requirement for field operation. In contrast, at a cement slurry:drilling fluid:spacer fluid ratio of 7:2:1, the mixed slurry exhibited a relatively high initial consistency that gradually decreased to below 10 Bc as the test proceeded, and the thickening time exceeded 300 min, fully meeting operational requirements. The thickening test involving the cement slurry mixed with spacer fluid also showed good compatibility, with a consistency below 10 Bc throughout the test and slurry stability established beyond 300 min.

These results confirm a successful liner cementing operation in this well. The proposed anti-contamination agent can be effectively applied under field conditions and may contribute significantly to future advanced cementing operations.

## 5. Conclusions

(1) This article combined the co-precipitation method with the ion exchange method to introduce aminotrimethylene phosphonic acid (ATMP) into the interlayer of zinc aluminum hydrotalcite (Zn/Al LDH) for the first time. Characterization techniques such as XRD, FT-IR, SEM, etc. show that the new drilling fluid anti-pollution agent Zn/Al TMP LDH was successfully prepared.

(2) The anti-pollution agent has good compatibility with common isolation liquid materials, and the prepared anti-pollution isolation liquid still exhibits good rheological characteristics after aging at 187 °C and 145 MPa. The isolation fluid prepared with the anti-pollution agent as the core is chemically stable and compatible with cement slurry and drilling fluid after mixing. It still has good rheological properties after curing at 187 °C × 145 MPa × 120 min. When added at a dosage of 5 wt%, it can effectively improve the contact pollution between cement slurry and drilling fluid, meeting the construction requirements.

(3) The isolation fluid prepared using this anti-pollution agent was successfully applied in the cementing operation of a well in a high grinding block. The isolation fluid effectively solves the problem of contact pollution between cement slurry and drilling fluid, indicating that the anti-pollution agent has practical application value.

## 6. Limitations and Uncertainties

Although this study confirms the excellent potential of zinc/aluminum amino trimethylene phosphonic acid layered double hydroxide (Zn/Al ATMP LDH) as an anti-pollution additive, there are still several uncertainties and research limitations. Firstly, all thickening time tests and rheological performance tests were conducted in a controlled laboratory environment; Fluctuations in temperature (±5 °C), pressure (±10 megapascals), and mixing efficiency during on-site operating conditions may cause deviations in the actual effectiveness of the product. Secondly, the anti-pollution mechanism proposed in this article (Figure 9) is mainly derived from indirect characterization methods such as X-ray diffraction (XRD), Fourier-transform infrared spectroscopy (FT-IR), thermogravimetric–differential thermogravimetric analysis (TG-DTG), and macroscopic performance data. However, this study has not yet obtained direct experimental evidence of the release kinetics of aminotrimethylphosphonic acid (ATMP) and the calcium ion chelation process at the molecular level (such as in situ spectroscopic testing and real-time monitoring data of ion-selective electrodes). Direct performance comparison tests have not yet been conducted between this material and commercial-scale inhibitors or other layered double hydroxide (LDH)-based materials under the same experimental conditions.

## Figures and Tables

**Table 1:** SD series theme raw material CAS.

Name	Function	CAS No. of Key Raw Materials
SDP-1	Dispersant	Acetone: 67-64-1; formaldehyde: 50-00-0; sodium naphthalene sulfonate condensate: 9084-06-4
SD210	Retarder	2-acrylamido-2-methylpropane sulfonic acid (AMPS): 15214-89-4; acrylic acid: 79-10-7; itaconic acid: 97-65-4
SD130	Fluid loss agent	AMPS: 15214-89-4; acrylamide: 79-06-1
SD52	Defoamer	Polydimethylsiloxane: 63148-62-9
SD77	Enhancer/anti-gas channeling agent	Silica fume: 69012-64-2; styrene–butadiene latex: 9003-55-8
SD35	Suspension stabilizer	Hydroxyethyl cellulose (HEC): 9004-62-0; polyacrylate: 9003-03-6

**Table 2:** Synthetic raw material CAS.

Full Chemical Name	CAS	English Name
ATMP	6419-19-8	Aminotris (methylenephosphonic acid)
Zn(NO_3_)_2_·6H_2_O	10196-18-6	Zinc nitrate hexahydrate
Al(NO_3_)_3_·9H_2_O	7784-27-2	Aluminum nitrate nonahydrate
NaOH	1310-73-2	Sodium hydroxide
NaNO_3_	7631-99-4	Sodium nitrate

**Table 3:** Compatibility of the anti-contamination agent in spacer fluid systems.

Spacer Fluid Formulation	Dispersant	Fluid Loss Additive	Suspension Stabilizer	Aging Conditions	Aging Time/min
Formulation 1	D1	L1	S1	187 °C, 145 MPa, 120 min	180
Formulation 2	D2	L2	S2	187 °C, 145 MPa, 120 min	180
Formulation 3	D1	L2	S3	187 °C, 145 MPa, 120 min	180
Formulation 4	D2	L1	S4	187 °C, 145 MPa, 120 min	180

**Note:** The base spacer fluid formulation was water + 4–6% suspension stabilizer + 5% anti-contamination agent + 2–3% dispersant + 0.2–0.6% fluid loss additive + 10% micro-manganese + 50% barite.

**Table 4:** Rheological properties of the anti-contamination spacer fluid before aging.

Spacer Fluid Formulation	Rotational Speed (r/min)						AV/mPa·s	YP/lb/100 ft^2^	K/Pa·s^n^	n
	3	6	100	200	300	600				
Formulation 1	6	9	54	78	96	140	70	52	1.87	0.52
Formulation 2	2	4	25	40	52	81	40.5	23	0.42	0.67
Formulation 3	5	10	31	42	51	74	37	28	1.54	0.45
Formulation 4	2	4	12	21	31	50	25	12	0.86	0.07

**Table 5:** Rheological properties of the anti-contamination spacer fluid after aging.

Spacer Fluid Formulation	Rotational Speed (r/min)						AV/mPa·s	YP/lb/100 ft^2^	K/Pa·s^n^	n
	3	6	100	200	300	600				
Formulation 1	7	9	61	94	122	174	87.0	70	1.22	0.63
Formulation 2	2	3	31	57	80	136	68.0	24	0.19	0.86
Formulation 3	1	2	11	19	29	49	24.5	9	0.06	0.88
Formulation 4	1	2	10	17	25	43	21.5	7	0.07	0.83

**Table 6:** Rheological test results for the mixed slurry with a drilling fluid:spacer fluid ratio of 7:3 at different temperatures.

Drilling Fluid:Spacer Fluid = 7:3	Rotational Speed (r/min)						AV/mPa·s	YP/lb/100 ft^2^	K/Pa·s^n^	n
	3	6	100	200	300	600				
50 °C	8	11	37	57	75	120	60	30	0.69	0.64
130 °C; 24 h	6	10	45	67	78	126	63	30	1.76	0.50
130 °C; 48 h	4	5	32	55	79	126	63	32	0.24	0.82
140 °C; 24 h	9	11	39	74	83	142	71	24	0.58	0.69
140 °C; 48 h	8	15	36	65	78	116	58	40	0.49	0.70
150 °C; 24 h	3	7	34	56	63	93	46.5	33	0.97	0.56
150 °C; 48 h	4	7	48	74	99	161	80.5	37	0.83	0.66

**Table 7:** Rheological test results for the mixed slurry with a drilling fluid:spacer fluid ratio of 5:5 at different temperatures.

Drilling Fluid:Spacer Fluid = 5:5	Rotational Speed (r/min)						AV/mPa·s	YP/lb/100 ft^2^	K/Pa·s^n^	n
	3	6	100	200	300	600				
50 °C	11	15	48	69	89	141	70.5	37	1.37	0.56
130 °C; 24 h	11	16	48	75	95	167	83.5	23	1.01	0.62
130 °C; 48 h	6	8	45	58	84	140	70	28	1.24	0.57
140 °C; 24 h	6	9	45	66	95	139	69.5	51	0.70	0.68
140 °C; 48 h	7	9	46	69	97	155	77.5	39	0.72	0.68
150 °C; 24 h	26	48	154	173	195	263	131.5	127	26.09	0.21
150 °C; 48 h	2	5	44	68	94	131	65.5	57	0.65	0.69

**Table 8:** Rheological test results for the mixed slurry with a drilling fluid:spacer fluid ratio of 3:7 at different temperatures.

Drilling Fluid:Spacer Fluid = 5:5	Rotational Speed (r/min)						AV/mPa·s	YP/lb/100 ft^2^	K/Pa·s^n^	n
	3	6	100	200	300	600				
50 °C	9	17	57	84	108	169	84.5	47	1.47	0.58
130 °C; 24 h	6	14	53	75	96	147	73.5	45	1.68	0.54
130 °C; 48 h	4	7	22	46	60	105	52.5	15	0.10	0.91
140 °C; 24 h	11	20	45	67	84	139	69.5	29	1.24	0.57
140 °C; 48 h	10	15	60	95	120	188	94	52	1.20	0.63
150 °C; 24 h	4	6	38	64	80	126	63	34	0.60	0.68
150 °C; 48 h	3	5	38	60	76	112	56	40	0.76	0.63

## Data Availability

The original contributions presented in this study are included in this article. Further inquiries can be directed to the corresponding author.
